# Beta Blocker Therapy in Heart Failure Patients with Active Cocaine Use: A Systematic Review

**DOI:** 10.1155/2020/1985379

**Published:** 2020-05-08

**Authors:** Baldeep K. Mann, Janpreet S. Bhandohal, Mohammad Saeed, Gerald Pekler

**Affiliations:** ^1^Department of Internal Medicine, New York Medical College, Metropolitan Hospital Center, New York, NY 10029, USA; ^2^Department of Internal Medicine, UCLA, Kern Medical Center, Bakersfield, CA 93306, USA; ^3^Division of Cardiology, New York Medical College, Metropolitan Hospital Center, New York, NY 10029, USA

## Abstract

**Background:**

Cocaine use is associated with multiple cardiovascular complications including heart failure. The use of different types of beta blockers in heart failure patients with active cocaine use is still a matter of debate. In this review, our objective is to systematically review the available literature regarding the use of beta blockers in the treatment of heart failure patients with concurrent cocaine use.

**Methods:**

PubMed, EMBASE, Web of Science, and Clinical Trials.gov were searched from inception to March 2019 using the Medical Subject Headings (MeSH) terms “cocaine”, “heart failure”, “beta blocker,” and “cardiomyopathy”. Only studies containing the outcomes of heart failure patients with active cocaine use who were treated with beta blockers were included.

**Results:**

The search resulted in 2072 articles out of which 12 were finally included in the review. A total number of participants were 1994 with a median sample size of 111. Most of the studies were retrospective in nature with Oxford Centre for Evidence-Based Medicine (OCEBM) Levels of Evidence from 3 to 5. The main primary outcomes included readmission rates, mortality, left ventricular ejection fraction (LVEF) improvement, New York Heart Association (NYHA) functional class, and major adverse cardiovascular events (MACEs). In the studies analyzed, beta blockers were found to have either a beneficial or a neutral effect on primary outcomes in heart failure patients with active cocaine use.

**Conclusion:**

The use of beta blocker therapy appears to be safe and beneficial in heart failure patients with active cocaine use, although the evidence is not robust. Furthermore, large-scale studies are required to confirm this finding.

## 1. Introduction

Cocaine is a naturally occurring alkaloid which was first isolated in 1860 from the leaves of *Erythroxylum coca* [1]. It can produce various deleterious cardiovascular effects through different mechanisms including hypertension, aortic dissection, myocardial ischemia or infarction, cardiomyopathy or heart failure, dysrhythmias, pulmonary hypertension, and stroke [2]. Intracoronary infusions of cocaine have caused acute elevation in left ventricle (LV) pressures, LV dilatation, and reduction in contractility in both animal and human experimental models [3, 4]. Left ventricular dysfunction and chronic heart failure (HF) have been reported in cocaine users without evidence of ischemic heart disease [5]. Beta blockers are a class I American College of Cardiology/American Heart Association (ACC/AHA)-sanctioned treatment for heart failure, and reluctance to use them in cocaine-using patients is based on the dogma of unopposed alpha-stimulation, an inconsistent, unpredictable, and rare phenomenon [6, 7]. According to the 2014 ACC/AHA guidelines, beta blockers appear to be safe in patients of non-ST elevation acute coronary syndrome (ACS) with recent cocaine use. Beta blockers should not be administered in patients with signs of acute cocaine toxicity due to risk of potentiating coronary spasm (Class III, Level of Evidence C) [8]. In addition, there are no clear guidelines on the use of beta blockers in heart failure patients with active cocaine use. To our knowledge, there is no existing systematic review on this subject in the literature. In this review, our objective is to assess the available literature on the use of beta blocker therapy in patients with heart failure and active cocaine use.

## 2. Methods

This systematic review adhered to the “Preferred Reporting Items for Systematic Reviews and Meta-Analyses statement” (Supplement 1) [9]. Studies were considered for inclusion if they met the following criteria: human studies, heart failure patients with active cocaine use, interventions including beta blocker (cardioselective and noncardioselective) treatment, outcomes including hospital readmission rates, major cardiovascular events, all-cause mortality, New York Heart Association (NYHA) functional class, and left ventricular ejection fraction (LVEF). Studies not involving heart failure patients with active cocaine use being treated with beta blockers were excluded from our review.

An electronic literature search was performed for articles published in the databases PubMed, EMBASE, Web of Science, and Clinical Trials.gov from inception to March 2019 using the Medical Subject Headings (MeSH) terms “cocaine”, “heart failure”, “beta blocker,” and “cardiomyopathy”. MeSH terms were combined as “cocaine” and “heart failure”, “cocaine” and “cardiomyopathy”, “cocaine” and “heart failure” and “beta blocker”, “cocaine” and “cardiomyopathy,” and “beta blocker” to obtain a maximum number of articles. No additional records were found through individual search of references. The search was limited to humans and English language (including articles in other languages with English translation).

**Figure 1:**
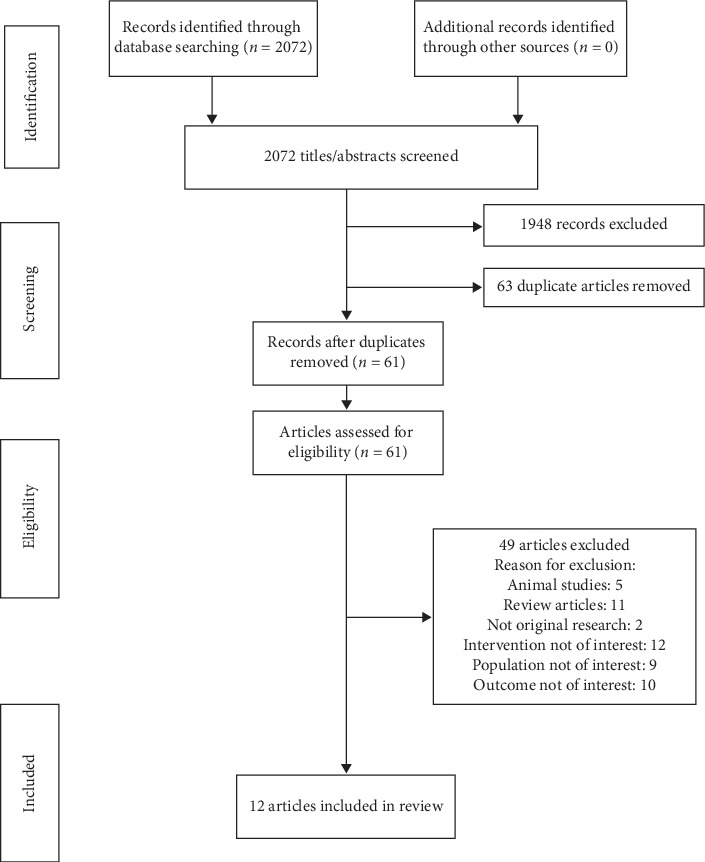
Preferred Reporting Items for Systematic Reviews and Meta-Analyses (PRISMA) flow diagram of articles included in systematic review.

The intersection of all searches yielded 2072 articles. After applying the inclusion and exclusion criteria, 12 articles were included in our final review (Figure 1). The final articles included were reviewed in detail by three investigators. Data extracted from each article included type of publication, purpose, design, time period, data source, method of identifying cases, sample size, follow-up period, clinical outcomes, type of readmission, and mortality (all-cause or disease specific, considering other comorbidities and organ dysfunction), and whether mortality was considered a separate or composite outcome. The statistical model of analysis was abstracted wherever available. Although it is unusual to include abstracts in a systematic review but due to the paucity of data in this field, all available studies and articles listed in various journals that met the criteria were included such as abstracts, presentations, and case reports to draw final conclusions.

## 3. Results

A total of 12 articles met the inclusion criteria and were considered in our final review (Table 1). The most common reasons for excluding an article were (1) patients not receiving beta blockers during the course of treatment and follow-up and (2) not addressing the heart failure patients with cocaine use.

Publication dates ranged from 2010 through 2019. Table 1 displays the characteristics of the included articles. Of the 12 articles reviewed, ten were retrospective studies involving a review of medical records [10, 11, 13, 15–21], one study was a case series of four patients [12], and one was a case report [14]. The sample size ranged from 1 to 503 patients with a median of 111. The total number of patients was 1994. No timeline was defined for active cocaine use in any of the studies reviewed. The use of cocaine was confirmed by self-reported history and positive urine toxicology in six studies [10, 12, 16, 18, 20, 21], positive urine toxicology in another five [11, 13, 15, 17, 19], and only self-reported in one study [14]. Carvedilol (combined beta1,2 and alpha blocker) alone was used in five studies [11, 12, 14, 15, 21], carvedilol, labetalol (combined beta1,2 and alpha blocker), bisoprolol, metoprolol tartrate, and metoprolol succinate were used in one study [16], and carvedilol and metoprolol succinate were used in two studies [18, 20]. Both cardioselective and noncardioselective beta blockers (not specified) were used in one study [13], and three studies did not specify the type of beta blockers used [10, 17, 19]. The primary outcomes were readmission (all cause and heart failure related) in nine studies [10–13, 15–17, 19, 20], mortality in seven studies [10, 11, 13, 15–17, 19], LVEF improvement in six studies [12–14, 17, 18, 20], NYHA functional class in three studies [12, 18, 20], and major adverse cardiovascular events (MACEs) in four studies [16, 18, 20, 21] as shown in Table 2.

Nine studies provided either partial or complete data on statistical measures [10, 13, 15–21], and one study did not report any statistical outcomes [11]. Statistical analysis was not applicable for two studies (case series and case report) [12, 14]. The risk of bias within the studies was calculated using multivariate analysis in four studies [16, 19–21]. Due to the retrospective nature of studies, variance in analytic approaches, and incomplete statistical data (also because of only abstract publication), formal synthesis was not possible and risk of bias across studies could not be determined. Four original studies included had difference in statistical design and/or primary outcomes limiting the opportunity for meta-analysis [16, 18–20]. Table 2 summarizes the measured statistical outcomes of studies.

Among the outcomes supported by statistical data, five studies compared HF patients with cocaine use on beta blockers against HF patients with cocaine use without beta blockers [13, 15, 19–21]. Out of these five studies, two reported no difference in myocardial infarction (MI) occurrence [13, 15], no difference in one-year mortality in two studies [15, 19], reduced HF-related readmission in two studies [19, 20], improved NYHA functional class and LVEF in one study [20], and lower MACE in one study [21]. Two studies compared HF patients with concurrent beta blockers and cocaine use against HF patients with beta blockers without cocaine use [10, 16]. Higher readmission rates among cocaine users were found in one study but mentioned that the social, economic, or therapeutic factors may be contributors along with physiologic consequences of cocaine [10]. Another study did not show any difference in HF-related readmissions, MACE, or mortality [16]. Two studies had no control group [17, 18]. Out of these, one study reported no significant change in LVEF [17] and one reported improved NYHA functional class and LVEF, but the sample size was small and follow-up was shorter comparatively [18].

## 4. Discussion

Cocaine can induce ischemic and nonischemic cardiomyopathies through different mechanisms. Although, myocardial infarction and scarring is considered a principal cause for LV dysfunction in cocaine abusers, exposure can cause acute and chronic reduction in LV contractility without ischemic heart disease. A condition known as cocaine-induced adrenergic surge similar to Takotsubo cardiomyopathy and pheochromocytoma-induced cardiomyopathy can occur, which is underlying pathophysiology behind these findings [2]. The other mechanisms by which cocaine can cause myocardial dysfunction include impaired intracellular calcium handling (due to local anesthetic properties of cocaine inducing a negative inotropic effect), myocyte apoptosis, elevated levels of reactive oxygen species, and eosinophilic myocarditis [2]. Beta blockers are considered a lifesaving therapy in ischemic heart disease and ischemic and nonischemic cardiomyopathy as they help in attenuating the myocardial oxygen demand and hyperadrenergic state [2]. There are no clear recommendations for the use of beta blocker therapy in active cocaine users with heart failure. According to the 2013 American College of Cardiology Foundation/American Heart Association (ACCF/AHA) guidelines for management of heart failure, the safety and efficacy of beta blockers for chronic HF due to cocaine use are unknown [22].

In this review, we included articles that evaluated the effect of beta blockers in heart failure patients actively using cocaine. The use of beta blockers in patients with cocaine-induced chest pain has been tested in several studies, but the data regarding their utility in heart failure patients using cocaine are very limited. There are no large-scale studies or randomized controlled trials available on this topic. Our search found only small-scale retrospective studies, and all except one (Finks et al. [15]) were single centered. The primary outcomes and statistical analysis (wherever available) are presented in Table 2. The level of evidence across all the studies ranged from 3 to 5 as per the Oxford Centre for Evidence-Based Medicine (Table 2) [23]. In the studies reviewed, beta blocker therapy (both selective and nonselective) was found to have beneficial effects and no additional adverse events in cocaine users. Within the literature we reviewed, there was no evidence of detrimental effects of beta blocker therapy in this group of patients.

Upon further analysis of studies defining the type of beta blockers used in patients with active cocaine use, a total of 882 patients were identified across these studies who were prescribed any type of beta blockers [11, 12, 14–16, 18–21]. The majority of these patients were prescribed carvedilol (710, 80.5%), twenty-six (3%) received either carvedilol or labetalol (not specified), and the rest (146, 16.5%) received other types of beta blockers including cardioselective and noncardioselective beta blockers. Ninety patients (10.2%) received cardioselective (metoprolol or bisoprolol) beta blockers. Although the outcomes were similar in patients receiving carvedilol and other beta blockers, it would be premature to say that the efficacy and safety are equivalent between different beta blocker classes due to large variation in sample size. The dose of beta blockers was determined as per clinical discretion and was not specified in any of the articles except two (Littmann et al. [12] and Ocal et al. [14]) which were a case series and a case report, respectively. Littmann et al. [12] titrated four patients to a maximum of carvedilol 25 mg twice daily while Ocal et al. [14] used carvedilol 6.25 mg twice daily in one patient. None of the larger studies reported the dose of beta blockers. Therefore, the ideal dose for safety and efficacy cannot be determined.

Most of the studies included patients with heart failure with reduced ejection fraction (HFrEF) and had a follow-up period of one year or less (Table 2). One study (Alvi et al. [21]) also included patients with heart failure with borderline ejection fraction (HFbEF) and heart failure with preserved ejection fraction (HFpEF). In studies defining the age group of patients, mostly were in midfifties [12, 15–20]. As a result, outcomes cannot be generalized to other categories of heart failure and age groups. Among the articles included, four were full publications of original studies [16, 18–20]. All studies were retrospective reviews of medical records at a single center. A total number of patients receiving beta blockers while on cocaine were 397 across these studies. Most of the patients were males (331, 83%). Three studies reported race, the majority were African American (111 out of 166, 67%) [16, 18, 20]. The longest follow-up period up to 4000 days was observed in a study by Nguyen et al. [16], the rest of the studies had a follow-up of 12 months [18–20]. As a result of this disparity in patient selection in terms of race and gender, application of outcomes outside these demographic categories is uncertain. Moreover, the shorter follow-up period in most studies questions the long-term safety of beta blocker intervention.

This review has several limitations worth noting. First, there is scarcity of research done on this topic which may lead to bias in their results. To decrease this risk, we included abstracts, presentations, case series, and case reports in our review. Second, due to the retrospective nature of the studies and small sample size, the level of evidence is weak. Furthermore, there is incomplete reporting of the statistical analysis among the studies. Third, since the studies did not report the complete statistical analysis, the risk of bias is high, and the formal synthesis of results could not be performed. Due to the heterogenicity in the study designs (case control or retrospective cohort), the central values could not be compared. Finally, patient population overlap could not be excluded in studies with the same author as there appears to be a similar pool of data (Finks et al. [11, 15], Egbuche et al. [17, 19], and Lopez et al. [18, 20]).

## 5. Conclusions

In our systematic review of the literature, we concluded that the evidence supporting the benefit and effectiveness of beta blocker therapy in treating heart failure patients with active cocaine use is weak. Although the use of beta blockers was not associated with any major adverse cardiovascular events, it is unclear whether other classes of beta blockers are as effective and safe as carvedilol (combined alpha and beta blocker) which was used in the majority of patients. Further large-scale studies and randomized controlled trials are needed to confirm these findings. This could be challenging because of the under reporting of cocaine use by patients and poor compliance with treatment and follow-up among cocaine users. In addition, regular facilitation of cocaine cessation by health care providers in these patients could lead to exclusion from the study group during the follow-up period. Therefore, observational studies on large scale could be a more reasonable alternative. The studies should clearly state the temporal association of cocaine use and beta blocker administration, the type of beta blocker (cardioselective vs noncardioselective) used along with the dose, primary end points, statistical analysis used and follow-up period. So far, the available evidence suggests that beta blockers, especially carvedilol, should be used in heart failure patients (especially HFrEF) with concurrent cocaine use who meet the criteria.

## Figures and Tables

**Table 1:** Characteristics of studies examining the effect of beta blockers in heart failure patients with active cocaine use.

Source	Article type	Study type	No. of patients	Age group (years) of cases	Heart failure category	Beta blocker class
Ahmed et al. [10]	Meeting abstract	Retrospective cohort (2008)	326	Not specified	Not specified	Not specified
Finks et al. [11]	Conference abstract	Case control (72 months)	76	Not specified	HFrEF	Combined *α* and *β* blocker (carvedilol) and other *β* blockers (not specified)
Littmann et al. [12]	Letter to the editor	Case series	4	51–57	HFrEF	Combined *α* and *β* blocker (carvedilol)
Akpa et al. [13]	Conference abstract	Case control (2006–2008)	132	Not specified	HFrEF	Cardioselective and noncardioselective (not specified)
Ocal et al. [14]	Case study	Case report	1	34	HFrEF	Combined *α* and *β* blocker (carvedilol)
Finks et al. [15]	Conference abstract	Case control (72 months)	217	57.3 (SD 4.8)	HFrEF	Combined *α* and *β* blocker (carvedilol)
Nguyen et al. [16]	Original study publication	Retrospective cohort (1993–2012)	267	55.5 (SD 7.51)	Not specified (LVEF <45%)	Cardioselective (bisoprolol, metoprolol) and noncardioselective (carvedilol, labetalol)
Egbuche et al. [17]	Meeting abstract	Retrospective cohort	90	56.1(SD 7.8)	HFrEF	Not specified
Lopez et al. [18]	Original study publication	Retrospective cohort (01/2010–06/2016)	38	54 (SD 8.4)	HFrEF	Combined *α* and *β* blocker (carvedilol) and cardioselective *β* blocker (metoprolol)
Egbuche et al. [19]	Original study publication	Retrospective cohort (2011–2014)	268	54.1 (SD 7)	HFrEF	Combined *α* and *β* blocker (carvedilol), cardioselective *β* blocker (metoprolol), and mixed type
Lopez et al. [20]	Original study publication	Retrospective cohort	72	54 (SD 8.4)	HFrEF	Combined *α* and *β* blocker (carvedilol) and cardioselective *β* blocker (metoprolol)
Alvi et al. [21]	Meeting abstract	Retrospective cohort	503	Not specified	HFrEF, HFbEF, and HFpEF	Combined *α* and *β* blocker (carvedilol)

HFrEF, heart failure with reduced ejection fraction; HFbEF, heart failure with borderline ejection fraction; HFpEF, heart failure with preserved ejection fraction; LVEF, left ventricular ejection fraction; SD, standard deviation.

**Table 2:** Study outcomes and conclusions along with the level of evidence as per the Oxford Centre for Evidence-Based Medicine 2011 Levels of Evidence.

Source	Study outcomes tested	Follow-up period	Study outcomes (statistical analysis)	Conclusion	Level of evidence [23]
Ahmed et al. [10]	Readmission rates and mortality	6 months	Readmission rates (HR, 1.8; 95% CI, 1.16–2.7; *p*=0.02)	Readmission rate higher in cocaine users. No difference in mortality	3

Finks et al. [11]	ED care, all-cause ED and readmission rates, length of stay, recurrent MI, and mortality	Not provided	Not provided	Carvedilol in patients with cocaine-induced chest pain and heart failure was safe	4

Littmann et al. [12]	Readmission rates, LVEF, and NYHA class	6–13 months	Not applicable	Carvedilol can improve LVEF and NYHA functional class in patients with ongoing cocaine use	4

Akpa et al. [13]	MI, ED visits, HF-related admissions, LVEF, mean BNP, and all-cause mortality	Not provided	MI occurrence (OR, 1.185; 95% CI, 0.277–5.069; *p*=0.819)	BB treatment of HFrEF with concomitant cocaine abuse may be safe	4

Ocal et al. [14]	LVEF	1 week	Not applicable	Successful treatment of cocaine-induced cardiotoxicity with carvedilol therapy	5

Finks et al. [15]	All-cause ED and readmission rates, recurrent MI rates, new onset HF, and mortality	6 months	ED visits (1.65 ± 1.88 vs 1.97 ± 2.39, *p*=NS), MI incidence (4.3% vs 3.6%, *p*=NS), HF occurrence (8.5% vs 8.6%, *p*=NS), One-year mortality (12.8% vs 10.0%, *p*=NS)	Carvedilol for cocaine-induced chest pain did not worsen 6-month outcomes in veterans with HFrEF and MI	4

Nguyen et al. [16]	HF readmissions, major adverse CV events, and death	4000 days	HF readmissions (HR, 0.66; 95% CI, 0.31–1.38), MACE (HR, 0.58; 95% CI, 0.27–1.09), Death (HR, 0.96; 95% CI, 0.39–2.34), All combined outcomes (HR, 0.76; 95% CI, 0.39–1.47), Mortality, cardioselective vs noncardioselective BB (HR, 1.50; 95% CI, 0.28–8.23)	BB therapy in systolic HF patients with cocaine use was not associated with adverse outcomes.	3

Egbuche et al. [17]	LVEF, readmission rates, and mortality	15.5 ± 8.6 months	Overall change in LVEF (1.9 ± 14.6, *p*=0.2), 39% patients with decreased LVEF (-10.6 ± 6.8),39% patients with increased LVEF (14.3 ± 7.5), 22% patients with no change in LVEF, and average rehospitalizations (3.2 ± 3.3)	Continuous BB therapy in HFrEF patients with cocaine abuse has variable effects on LVEF	3

Lopez et al. [18]	NYHA functional class, LVEF, and major adverse CV events	12 months	NYHA (*S* = –108, *p* < 0.0001) LVEF (*S* = 141.5, *p* < 0.0001)	BB therapy in cocaine users with HFrEF is associated with lower NYHA class and higher LVEF. No MACE was observed.	3

Egbuche et al. [19]	30-day all-cause readmissions, HF-related readmissions, and mortality	12 months	All-cause readmissions, 30 days (OR, 0.19; 95% CI, 0.06–0.64; *p*=0.007), HF-related readmissions, 30 days (OR, 0.17; 95% CI, 0.05–0.56; *p*=0.004), One-year mortality (OR, 0.88; 95% CI, 0.17–7.19; *p*=0.91)	BB therapy reduces 30-day readmission rate but not one-year mortality in HFrEF patients with concurrent cocaine use	3

Lopez et al. [20]	NYHA functional class, LVEF, CRCE, and HF readmissions	12 months	Improvement in NYHA functional class (RR, 2.24; 95% CI, 1.14–4.41; *p*=0.0106), Improvement in LVEF (RR, 2.46; 95% CI, 1.27–4.78; *p*=0.0031), CRCE (OR, 1.21; 95% CI, 1.04–1.42; *p*=0.0031), HF-related readmission (RR, 0.15; 95% CI, 0.02–1.18; *p*=0.0383)	BB therapy is associated with improvement in NYHA functional class and LVEF, lower incidence of CRCE, and HF-related readmissions in HFrEF patients with active cocaine use.	3

Alvi et al. [21]	Major adverse CV events	720 days	MACE similar in entire cohort (32% vs 38%, *p*=0.26), HFpEF (30% vs 33%, *p*=0.67), HFbEF (32% vs 36%, *p*=0.70), MACE lower in HFrEF (34% vs 47%, *p*=0.04), and lower MACE in multivariate model (HR, 0.66; 95% CI, 0.481–0.863)	Carvedilol is safe and may be effective among HF patients who use cocaine	3

BB, beta blocker; BNP, brain natriuretic peptide; CI, confidence interval; CRCE, cocaine-related cardiovascular events; CV, cardiovascular; ED, emergency department; HF, heart failure; HFrEF, heart failure with reduced ejection fraction; HFbEF, heart failure with borderline ejection fraction; HFpEF, heart failure with preserved ejection fraction; HR, hazard ratio; LVEF, left ventricular ejection fraction; MACE, major adverse cardiovascular events; MI, myocardial infarction; NS, nonsignificant; NYHA, New York Heart Association; OR, odds ratio; RR, relative risk.
